# Destroyed by Fire

**Published:** 1856-01

**Authors:** 


*Destroyed by Fire.*—We learn, with sincere regret, that among the losses occasioned by the late fire in Syracuse, N. Y., was the *New York College of Dental Surgery,* occupying the third and fourth stories of the Wick's Block, as well as the dental rooms in the second story of Professor Westcott. The entire Museum, embracing, as we are informed, many valuable preparations, imported from Paris, were consumed, together with the apparatus and chemicals of the laboratory, the fixtures and apparatus of the lecture rooms. An extensive mineralogical and geological cabinet, belonging to Prof. W., and placed in the lecture room, were, in part, we are glad to learn, got out of the building. The paper from which we obtained the above particulars is unable to inform us whether the losses, or any part of them, sustained by Prof. W., were covered by insurance. We trust that this was the case.

